# Preparation of a Novel Polystyrene-Poly(hydroxamic Acid) Copolymer and Its Adsorption Properties for Rare Earth Metal Ions

**DOI:** 10.3390/polym12091905

**Published:** 2020-08-24

**Authors:** Xiaoyan Cao, Qing Wang, Shuai Wang, Ruilin Man

**Affiliations:** 1 College of Chemistry and Chemical Engineering, and Hunan Provincial Key Laboratory of Efficient and Clean Utilization of Manganese Resources, Central South University, Changsha 410083, China; caoxxyu@126.com (X.C.); 1900116@163.com (Q.W.); rlman@csu.edu.cn (R.M.); 2 School of Chemical and Environmental Engineering, and Jiangxi Province Engineering Research Center of Ecological Chemical Industry, Jiujiang University, Jiujiang 332005, China

**Keywords:** adsorption, hydroxamic acid resin, rare earth ions, separation, synthesis

## Abstract

In this study, a novel polystyrene-poly(hydroxamic acid) copolymer was synthesized as an effective adsorbent for the treatment of rare earth elements. Through the use of elemental analysis as well as FTIR, SEM, XPS, and Brunauer-Emmett-Teller (BET) surface area measurement, the synthesized polymer was found to have a specific surface area of 111.4 m^2^·g^−1^. The adsorption performances of rare metal ions were investigated under different pH levels, contact times, initial concentrations of rare earth ions, and temperatures. The adsorption equilibrium for La^3+^, Ce^3+^, and Y^3+^ onto a polystyrene-poly(hydroxamic acid) copolymer is described by the Langmuir model, which confirms the applicability of monolayer coverage of rare earth ions onto a polystyrene-poly(hydroxamic acid) copolymer. The amount of adsorption capacities for La^3+^, Ce^3+^, and Y^3+^ reached 1.27, 1.53, and 1.83 mmol·g^−1^ within four hours, respectively. The adsorption process was controlled by liquid film diffusion, particle diffusion, and chemical reaction simultaneously. The thermodynamic parameters, including the change of Gibbs free energy (∆*G*), the change of enthalpy (∆*H*), and the change of entropy (∆*S*), were determined. The results indicate that the adsorption of resins for La^3+^, Ce^3+^ and Y^3+^ was spontaneous and endothermic. The polymer was also used as a recyclable adsorbent by the desorption experiment.

## 1. Introduction

Rare earth elements, known as the vitamins of modern industry, are important non-recoverable strategic resources. Due to their unique properties, the global demand for rare earth elements and their compounds for use in many high-tech applications has dramatically increased [1,2,3,4]. China is endowed with abundant rare earth mineral resources, but most of them are confined to low-end mixed rare earth products [5,6]. To solve the problem of efficient enrichment and separation of rare earth elements, many studies have been conducted and methods developed, including redox precipitation [7,8], crystallization [9,10], ion exchange adsorption separation [11,12,13,14], and solvent extraction methods [15,16]. Of these methods, ion exchange adsorption separation technology is generally considered to be the most efficient method for separating rare earth elements.

Compared to the other methods, ion exchange is known for its simple operation, high separation efficiency, and reusable adsorbents. Accordingly, the ion exchange method is widely used in the enrichment and separation of rare earth elements. With the development of research, specialists have focused more on adsorption resins with high adsorption capacity, selectivity, and adsorption kinetics.

Hydroxamic acid and its derivatives contain RC(=O)NHOH groups that exhibit a strong ability to chelate metal ions. Commonly, one metal ion can chelate two or three hydroxamate molecules to form five-membered ring complexes (according to the valence of the metal sample). Notably, a large proportion of hydroxamic acids has been used as flotation collectors [17,18], analytical reagents [19], drugs [20], and chelating resins [21,22]. 

Based on the concept of hard and soft acids and bases (HSAB) [23,24,25], rare earth elements belonging to the hard acid category can easily form coordination bonds with hard bases or intermediate bases, which contain O, N, and S. Hydroxamic acid chelating resins have double ligands of hydroxyl and oxime groups. These ligands enable them to chelate with various metal ions and form stable heterocyclic complexes, providing good adsorption performances [22]. We introduced hydroxamic acid chelating resin for the enrichment and separation of rare earth elements. Hydroxamic acid resins are usually prepared by a grafting modification of the polyacrylate resin. However, the grafting of macromolecules leads to a lower hydroximation rate. In addition, copolymerization of hydroxamic acid cannot form spherical resin. Industrial resins are typically spherical because of spherical resin’s advantages, which include good filling performance, mechanical properties, and hydrodynamic properties. The copolymerization of polystyrene with hydroxamic acid not only forms spherical resin, but also possesses higher hydroxamic acid content. Moreover, this method can improve the adsorption performance and application performance of the resin at the same time.

In this study, a novel polystyrene-poly(hydroxamic acid) copolymer was first prepared by copolymerization of polystyrene with hydroxamic acid. Adsorption performances for extraction of La^3+^, Ce^3+^, and Y^3+^ were evaluated. The influence of experimental factors including contact time, effect of temperature, initial concentrations of metal ions, and pH on adsorption were investigated. The kinetic and thermodynamic properties of rare earth metal ion adsorption by the resin were studied. The kinetic and isothermal adsorption equations of resin were established. 

## 2. Experimental and Methods

### 2.1. Materials

The reagents used in this paper were purchased from the Aladdin Industrial Corporation (Shanghai, China) and the distilled water was prepared in lab. All of the reagents were analytically pure. Aqueous solutions of rare earth ions were prepared by dissolving La(NO_3_)_3_·6H_2_O, Ce(NO_3_)_3_·6H_2_O and Y(NO_3_)_3_·6H_2_O in distilled water, separately. The particle size of the resin was ground to 150–250 µm for characterization and adsorption tests.

### 2.2. Synthesis of the Resin

#### 2.2.1. Synthesis of Propyl Hydroxamic Acid

Certain proportions of sodium hydroxide and hydroxylamine hydrochloride were mixed in a 250 mL three-necked flask and reacted at 5 °C for half an hour. Then methyl acrylate was slowly added. After reaction under certain conditions (sodium hydroxide: hydroxylamine hydrochloride: ester = 1.5:1.2:1, reaction temperature = 35 °C, reaction time = 4 h), solution was obtained by acidification, filtration, and rotary evaporation.

#### 2.2.2. Synthesis of Polystyrene-Poly(hydroxamic Acid) Copolymer

According to certain proportions, styrene, propylene hydroxamic acid, and divinylbenzene were mixed evenly in a 250 mL three-necked flask. Then, the corresponding amount of benzoyl peroxide was added into the flask and stirred under certain conditions (temperature = 85 °C, reaction time = 1 h, 400 rpm) for pre-polymerization. Next, an aqueous solution containing polyvinyl alcohol (50 mL), liquid paraffin (8 g), and toluene (6 g) was added into the flask to react for 4 h. When yellow uniform pellets formed, the reaction temperature was increased to 95 °C for 2 h to ripen the resin.

#### 2.2.3. Purification of Polystyrene-Poly(hydroxamic Acid) Copolymer

Polystyrene-poly(hydroxamic acid) copolymer was extracted using petroleum ether at 85 °C for 3 h then washed with distilled water (or ethyl alcohol) repeatedly until the obtained polymer was free from the residual pore-forming agent. The polymer was then used as an ion exchanger. The particle size of the synthesized polymer was between 0.6 and 0.8 mm.

**Scheme 1:**
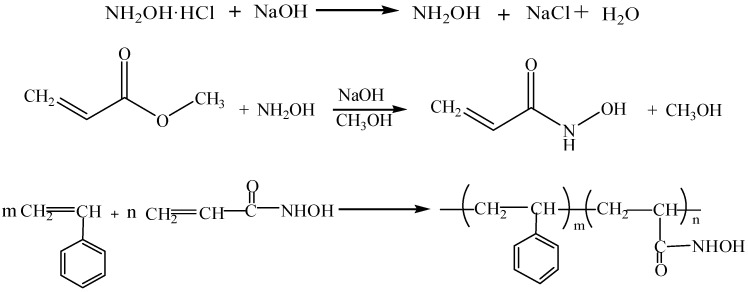
Synthetic route for the resin.

The synthetic route of the resin is represented in Scheme 1.

### 2.3. Characterization

The specific surface area was measured with N_2_ adsorption-desorption isotherms using the ASAP2020 (Micromeritics, Norcross, GA, USA) and Brunauer-Emmett-Teller (BET) methods. C, H, N, and O were analyzed using an Elemental Analyzer (Vario EL III, Elementa, Hanau, Germany). Fourier transform infrared spectroscopy (FTIR) (G510FTIR, Nicolet, Madison, WI, USA) provides complete molecular structure information, which facilitates the determination of chemical functional groups. The morphology of the resin was determined by scanning electron microscopy (SEM) (Mira3, Tescan, Brno, Czech Republic). The binding energy of the resin was confirmed by X-ray photoelectron spectrometer (XPS) (Escalab250Xi, Thermo Fisher, Waltham, MA, USA). 

### 2.4. Adsorption Experiments

Dry resin (0.2 g) and metal ion solution (25.0 mL, initial concentration was 0.01 mol·L^−1^) were mixed in a 100 mL conical flask. After being shaken at a constant temperature for an appropriate time, the solution was filtered. Then, the absorbance of metal ions in the filtrate was measured using an ultraviolet spectrophotometer. The adsorption capacity of the resin was calculated using the following equation:

(1) $$Q=\frac{(C_{0}-C_{t})V}{m}$$

where *Q* represents the adsorption capacity, *C*_0_ is the initial concentration of rare earth metal ions, *C_t_* is the rare earth metal concentration at *t* time, *V* is the volume of experiment solution and *m* is the mass of dry resin. 

### 2.5. Desorption Experiments

For desorption studies, 2 g of resin loaded with La(III), Ce(III), or Y(III) was mixed with 50 mL (2 mol·L^−1^) nitric acid. After being shaken at 30 °C for 4 h, the mixture was filtered and the rare earth ions in the aqueous solution were determined by atomic absorption spectrophotometer. The desorption efficiency of the resin was calculated using the following equation:

(2) $$R_{W}=\frac{C_{W}V_{W}}{Qm}\times 100\%$$

where *R_w_* (%) represents the desorption efficiency, *C_w_* (mol·L^−1^) is the concentrations of rare earth metal ions in eluent solutions, *V_w_* (L) is the volume of eluent, *Q* (mmol·g^−1^) is the adsorption capacity, and *m* (g) is the mass of dry resin.

## 3. Results and Discussion

### 3.1. Characterization 

#### 3.1.1. FTIR Study

**Figure 1:**
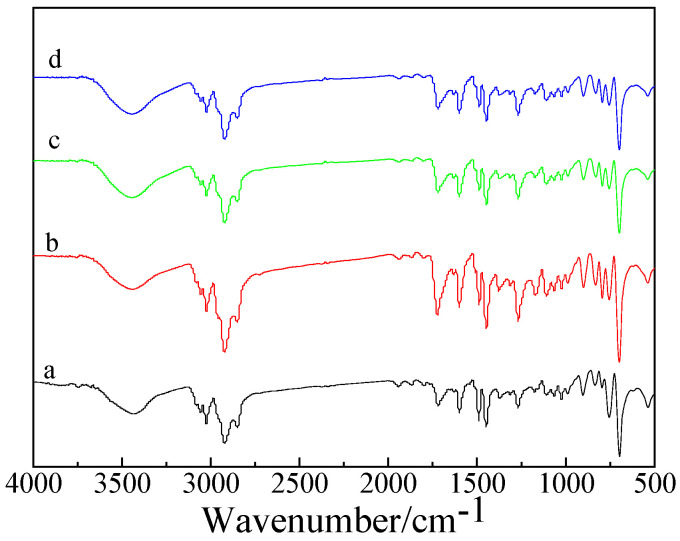
FTIR of the resin before (**a**) and after (**b**) La(III) or (**c**) Ce(III) or (**d**) Y(III) adsorption.

The FTIR spectra of the polystyrene-poly(hydroxamic acid) copolymer before and after treatment by La(III), Ce(III), and Y(III) are shown in Figure 1. As shown in Figure 1a, the N–H and O–H stretching bands of the resin appeared at around 3430 cm^−1^ and were assigned to the superposition peak of stretching vibrations [26]. The bands at around 3024 and 2921 cm^−1^ belonged to saturated C–H and unsaturated C–H stretching vibrations, respectively. The peaks at around 1719 and 1600 cm^−1^ were attributed to C=O [11] and benzene ring C=C stretching vibrations, respectively. In addition, the peaks were 1448 cm^−1^ for saturated C–H bending vibrations, 1269 cm^−1^ for single bond C–C stretching vibrations, and 697 cm^−1^ for benzene ring C–H bending vibrations. FTIR spectra showed that C=O and N–H stretching vibration peaks were apparent. Therefore, the desired product was synthesized by combining the results of elemental analysis. After adsorption of La(III), Ce(III), and Y(III), the peaks of N–H and O–H superposition shifted from 3430 cm^−1^ to a higher frequency nearby of 3445 cm^−1^, demonstrating an enhanced electron density of the resin. The difference between the FTIR spectra before and after adsorption suggested that there is coordination between the rare earth ions and the polystyrene-poly(hydroxamic acid) copolymer, and that the resin might react with rare earth ions through its N and O atoms in aqueous solutions.

#### 3.1.2. SEM Analysis

**Figure 2:**
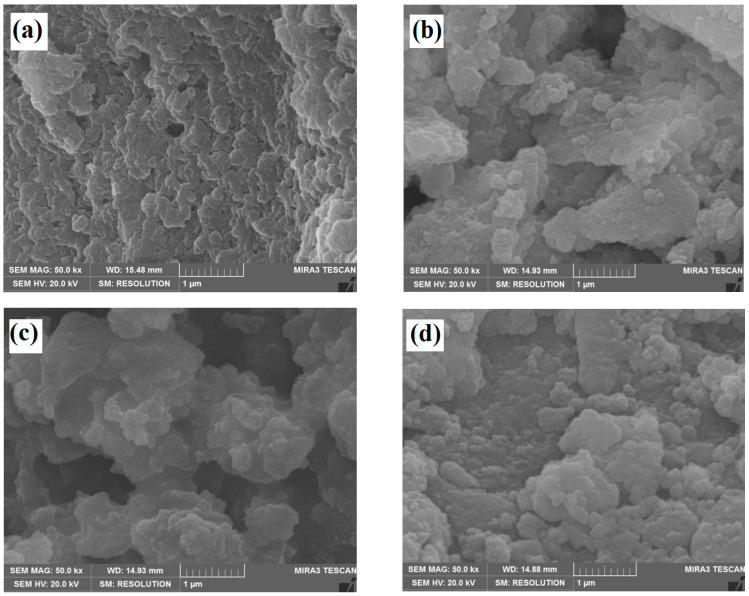
SEM images of the resin before (**a**) and after (**b**) La(III) or (**c**) Ce(III) or (**d**) Y(III) adsorption.

SEM analysis is an efficient method for observing morphology. The morphologies of the polystyrene-poly(hydroxamic acid) copolymer before and after adsorption of rare earth ions are presented in Figure 2. The surface of the resin was not flat and presented coarse pore structures, which may have contributed to the adsorption of metal ions. After being adsorbed, the morphology surfaces changed significantly. The structure and porosity of the resin changed, and a metallic luster appeared at the resin surface. We concluded that rare metal ions adsorbed on the surface of the resin. The BET surface area, average pore size, and pore volume for the resin were 111.4 m^2^·g^−1^, 5.93 nm, and 0.4869 cm^3^·g^−1^, respectively. The results demonstrated that the porous structure of the resin is mainly mesoporous. Elemental analysis showed that the nitrogen content of polystyrene-poly(hydroxamic acid) copolymer is 1.759%.

#### 3.1.3. XPS Analysis

**Figure 3:**
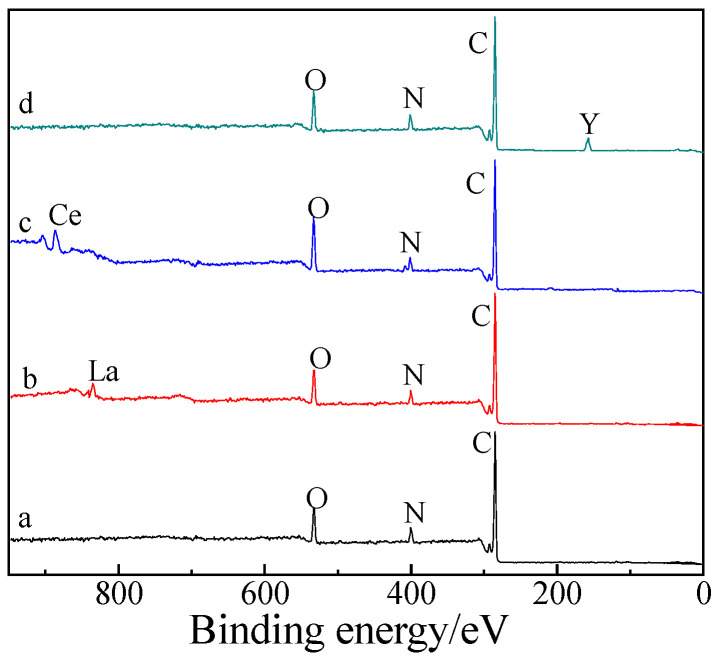
XPS spectra of the resin before (**a**) and after (**b**) La(III) or (**c**) Ce(III) or (**d**) Y(III) adsorption.

The surface composition and chemical state of the element were investigated by XPS. Figure 3 shows the full XPS spectrum of polystyrene-poly(hydroxamic acid) copolymer before and after treatment by La(III), Ce(III), and Y(III) over a binding energy range of 0~1200 eV. Compared with the spectrum before adsorption, new signals of La, Ce, and Y demonstrate the presence of La(III), Ce(III), and Y(III) cations on the resin, respectively. The binding energy of element nitrogen decreased and that of oxygen increased, which also indicated that there was coordination between the rare earth ions with polystyrene-poly(hydroxamic acid) copolymer and that rare metal ions were adsorbed on the surface of the resin. 

### 3.2. Adsorption Study

#### 3.2.1. Effect of Contact Time

**Figure 4:**
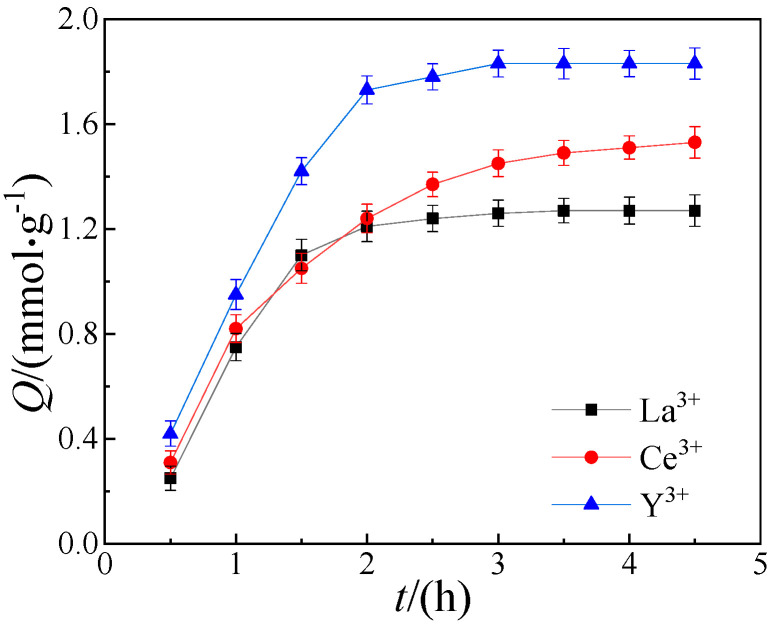
Effect of contact time on the resin (*C*_0_ = 0.01 mol·L^−1^; *T* = 40 °C pH for La^3+^, Ce^3+^, and Y^3+^ were 2.0, 1.0, and 3.0, respectively).

The effects of contact time on the adsorption of the resin for La^3+^, Ce^3+^, and Y^3+^ were investigated and the results are shown in Figure 4. It is clear that the adsorption capacities of rare earth ions increased with increases in contact time, reached equilibrium after around 3.5 h, 4 h and 3.5 h respectively, and remained steady the rest of the time. Remarkably, the amount of adsorption was obviously faster at the initial stage, potentially because, initially, the adsorbent site was vacant and the solute concentration gradient was high. Thus, it appeared that the adsorption capacity of rare earth ions on the resin was a rapid process [27], with equilibrium reached in four hours. Therefore, the contact time of four hours was considered appropriate in the following experiments.

#### 3.2.2. Effect of pH 

**Figure 5:**
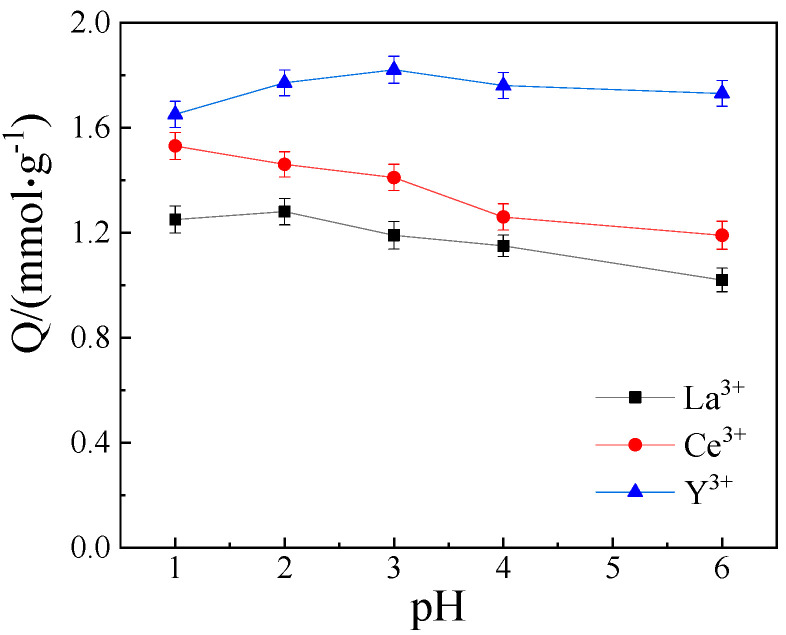
Effect of pH values on the resin (*C*_0_ = 0.01 mol·L^−1^, *t* = 4 h, *T* = 40 °C).

The effect of pH solution is an important parameter that influences the adsorption capacity of the adsorbent for metal ions [28,29]. Figure 5 shows the effect of pH on the adsorption of resin for La^3+^, Ce^3+^, and Y^3+^. Study at higher pH range was avoided since lanthanides tend to hydrolyze above pH 6 [11]. Therefore, the adsorption experiment was conducted at pH values from 1.0 to 6.0. As shown in Figure 5, the adsorption capacity for Y(III) increased with increasing pH, then decreased and tended to stabilize. The adsorption capacity for Ce(III) decreased with increasing pH and the adsorption trend of La(III) was the same as that of Ce(III). We concluded that the optimal pH levels for La^3+^, Ce^3+^, and Y^3+^ are 2.0, 1.0 and 3.0, respectively.

#### 3.2.3. Effect of Initial Concentration of Metal Ions

**Figure 6:**
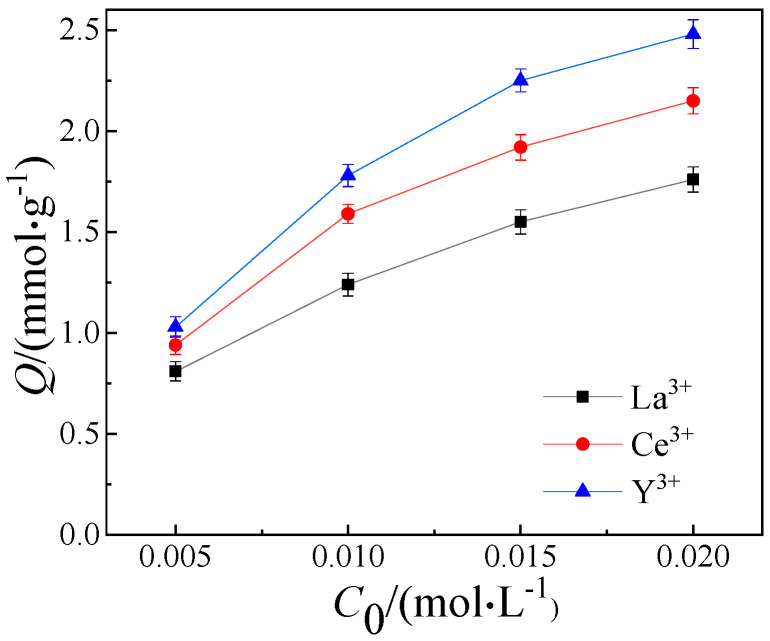
Effect of initial concentration of metal ions. (*t* = 4 h; *T* = 40 ℃; pH for La^3+^, Ce^3+^ and Y^3+^ were 2.0, 1.0 and 3.0 respectively).

The effect of initial concentration of metal ions is shown in Figure 6. The concentration of metal ions was evaluated in the range of 0.005~0.020 mol·L^−1^. The adsorption capacity of metal ions was sharply enhanced by increasing the rare earth ion concentration from 0.005 to 0.015 mol·L^−1^ and remained in a state of slow growth thereafter. This phenomenon could be interpreted as showing that the increase in the initial concentration can lead to an increase in the driving force of the adsorption process and can improve contact opportunities between metal ions and effective active sites in the solution [30]. With the increase of initial metal ion concentration, most of the adsorption sites became occupied and the adsorption efficiency began to be constant [26]. When the adsorption process reached the saturated adsorption capacity, the increase in ion concentration had less effect on adsorption capacity.

#### 3.2.4. Effect of Temperature

**Figure 7:**
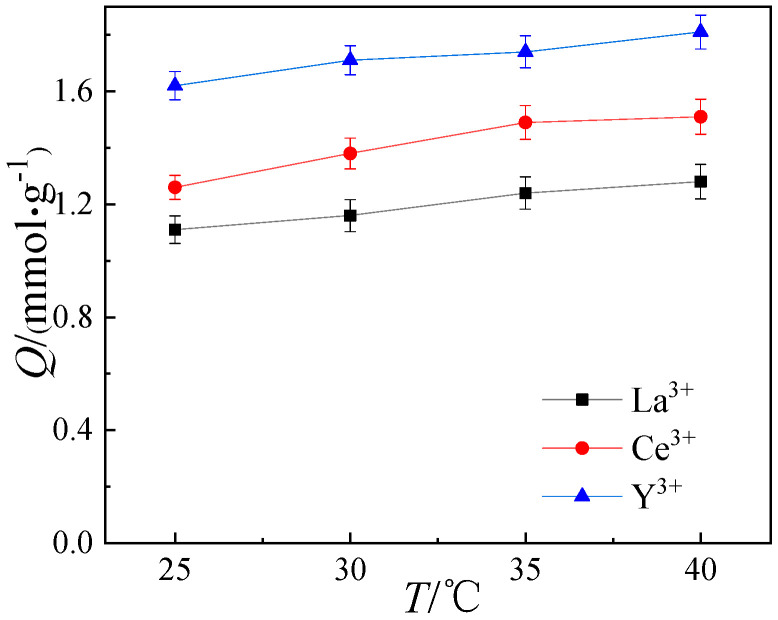
Effect of temperature. (*C*_0_ = 0.01 mol·L^−1^, *t* = 6 h; pH values for La^3+^, Ce^3+^, and Y^3+^ were 2.0, 1.0, and 3.0, respectively).

The effect of temperature on the adsorption of resin for La^3+^, Ce^3+^, and Y^3+^ was investigated and the results are shown in Figure 7. It can be seen that the adsorption capacity of metal ions increased with the temperature. However, the increase happened very slowly. This result showed that temperature has a lower effect on the adsorption capacity of metal ions. 

### 3.3. Adsorption Kinetics

The kinetic study was carried out to describe the reaction rate of the adsorption process and explore the adsorption mechanism. Adsorption kinetics data were fitted using liquid film diffusion, particle diffusion, and chemical reaction, which are expressed as Equations (3), (4), and (5) [31,32], respectively:

(3) $$-\ln\left(1-F\right)=k_{1}t$$

(4) $$1-3{\left(1-F\right)}^{2/3}+2(1-F)=k_{2}t$$

(5) $$1-{\left(1-F\right)}^{1/3}=k_{3}t$$

where *F = Q_t_ /Q*_e_*,* where *Q*_e_, and *Q_t_* are adsorption capacities (mmol·g^−1^) at equilibrium and at time *t* (min); and *k*_1_, *k*_2_*, k*_3_ denote the rate constants of the liquid film diffusion, particle diffusion, and chemical reaction (min^−1^), respectively.

**Figure 8:**
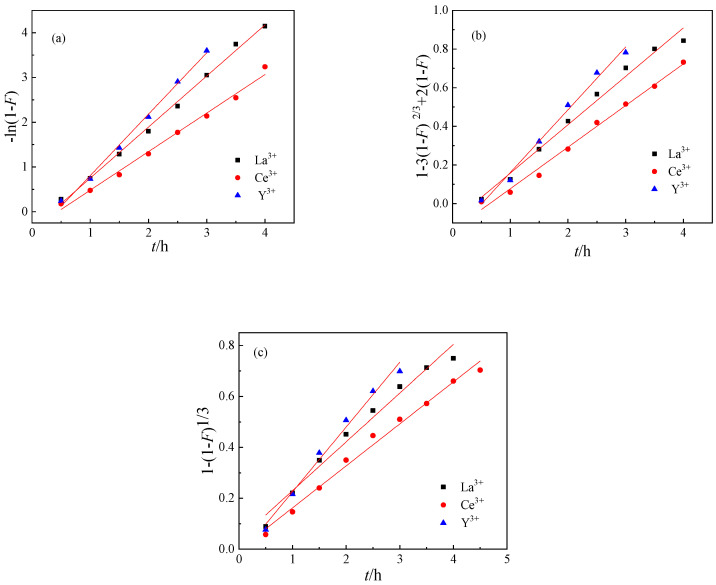
Linear forms of kinetic plots: (**a**) −ln(1 − *F*) vs. *t*; (**b**) 1 − 3(1 − *F*) ^2/3^ + 2(1 − *F*) vs. *t*; (**c**) 1 − (1 − *F*)^1/3^ vs. *t.*

Linear plots of −ln(1 − *F*) vs. *t*, 1 − 3(1 − *F*)^2/3^ + 2(1 − *F*) vs. *t* and 1 − 3(1 − *F*)^1/3^ vs. *t* are displayed in Figure 8. The constants calculated from the slope and intercept of those plots are given in Table 1. The values of the correlation coefficient (*R*^2^) of the three kinetics models were very high (close to 0.99). Based on the correlation coefficients, the three models match better with experimental data, suggesting that the adsorption process might be simultaneously controlled by liquid membrane diffusion, particle diffusion, and chemical reaction [33].

### 3.4. Adsorption Isotherm

**Figure 9:**
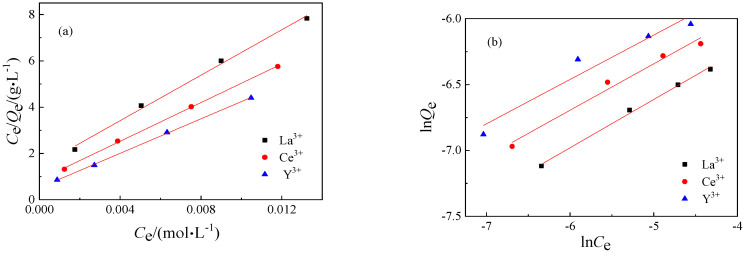
Linear forms of adsorption isotherm: (**a**) Langmuir and (**b**) Freundlich isotherms.

The adsorption isotherm reflects the variation of adsorption capacity compared to initial ion concentration at the same temperature. In this study, the experimental results obtained were tested by the Langmuir and Freundlich isotherms [34,35,36]. Their linear equations are expressed as follows:

(6) $$\frac{C_{e}}{Q_{e}}=\frac{C_{e}}{Q_{m}}+\frac{1}{Q_{m}K_{L}}$$

(7) $$\ln Q_{e}=\frac{1}{n}\ln C_{e}+\ln K_{F}$$

where *Q*_e_ (mmol·g^−1^) is the adsorption capacity at equilibrium, *Q*_m_ (mmol·g^−1^) is the maximum Langmuir adsorption capacity, *C*_e_ (mol·L^−1^) is the equilibrium concentration, *K*_L_ (L·mol^−1^) is the Langmuir constant, and n and *K*_F_ are Freundlich adsorption coefficients. The fitted results are shown in Figure 9 and the parameters are listed in Table 2.

Figure 9a shows that the equilibrium data were well fitted by the Langmuir isotherm. Moreover, the correlation coefficient (*R*^2^) of the Langmuir isotherm was much higher than that of the Freundlich isotherm, suggesting that the adsorption process of the resin to rare metal ions might be monolayer adsorption. Similar observations were reported for the adsorption of Ce(III) onto D151 resin [37].

### 3.5. Adsorption Thermodynamics

**Figure 10:**
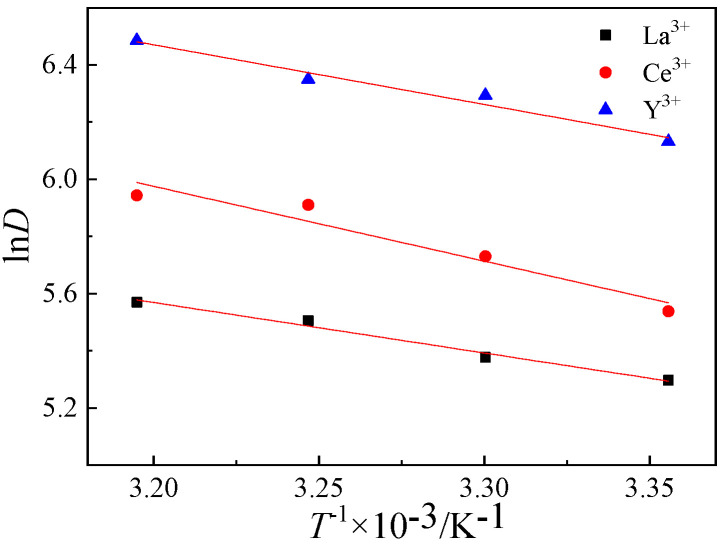
Linear fit plot of ln*D* vs. *T*^−1.^

Thermodynamic parameters are necessary to determine the spontaneity and heat exchange of adsorption. The thermodynamic parameters included change in Gibbs free energy (∆*G*), enthalpy (∆*H*), and entropy (∆*S*), which were determined using Equations (8)–(10) [38,39,40]. The fitted plots are shown in Figure 10 and the results are presented in Table 3.

(8) $$D=V(C_{0}-C_{e})/(mC_{e})$$

(9) $$\ln D=-\frac{\Delta H}{RT}+\frac{\Delta S}{R}$$

(10) $$\Delta S=\frac{\Delta H-\Delta G}{T}$$

where *D* is the distribution coefficient (L·g^−1^), *V* is the volume of solution (L), *m* is the mass of the resin (g), *R* is the gas constant (8.314 J·mol^−1^·K^−1^), and *T* stands for temperature (K). *C*_0_ (mol·L^−1^) and *C*_e_ (mol·L^−1^) are the initial concentration and equilibrium concentration of La(Ⅲ), Ce(Ⅲ), and Y(Ⅲ) ions, respectively.

As shown in Table 3, the enthalpy change (∆*H*) of the adsorption process was greater than zero, suggesting that the process was an endothermic reaction and the increase of temperature was beneficial to the adsorption, which is consistent with the previous conclusion. The negative ∆*G* values indicated that the adsorption was spontaneous. The positive ∆*S* indicated there was a high affinity between rare earth ions and the resin [41]. The adsorption was an endothermic chemical adsorption. 

### 3.6. Desorption Experiments

The regeneration of the adsorbent is an important factor required for practical application. In this study, desorption experiments were carried out and the results are listed in Table 4. After the resin desorption happened three times, desorption efficiency of the resin could reach 98.7%. We found that 1.6 mol ·L^−1^ nitric acid can be effectively desorbed from rare earth ion complexes, suggesting the obtained resin has good regeneration properties.

### 3.7. Comparison between Our Results and the References

In this study, the adsorption amount of polystyrene-poly(hydroxamic acid) resin was 1.27 mmol·g^−1^ for La(III), 1.53 mmol·g^−1^ for Ce(III), and 1.83 mmol·g^−1^ for Y(III). To evaluate the adsorption capacity of the synthesized polymer, some other adsorbent materials reported in previous studies for Ce(III) adsorption are listed in Table 5. Polystyrene-poly(hydroxamic acid) resin showed a significantly larger adsorption capacity for Ce(III) than most of the reported adsorbents. The synthesized resin exhibits considerable potential as an absorbent for rare earth ions.

## 4. Conclusions

In this paper, we reported the synthesis of polystyrene-poly(hydroxamic acid) copolymer by suspension polymerization. Based on our systematic characterizations and batch adsorption experimental results, the obtained polymer shows a large adsorption capacity for rare earth ions and the maximum adsorption capacities are 1.27 mmol·g^−1^ for La^3+^, 1.53 mmol·g^−1^ for Ce^3+^, and 1.83 mmol·g^−1^ for Y^3+^. The adsorption kinetics study indicated that the adsorption process of the resin for rare earth ions involves three stages: liquid membrane diffusion, particle diffusion, and chemical reaction. The Langmuir isotherm was more appropriate for fitting the equilibrium data than the Freundlich isotherm. The thermodynamics studies indicated that the adsorption process is spontaneous and endothermic. Spectral results implied that there is coordination between the rare earth ions with the polystyrene-poly(hydroxamic acid) copolymer and that chemical reactions might occur between rare earth ions and the N and O atoms of the polymer. In addition, the desorption tests proved that the obtained polymer can be used as a recyclable adsorbent. 

## Figures and Tables

**Table 1:** The kinetic parameters of different kinetic models.

Metal Ions	Fitting Models	Fitting Equations	*k*	*R* ^2^
La^3+^	liquid film diffusion	−ln(1 − *F*) = −0.3892 + 1.141*t*	1.1410	0.9956
	particle diffusion	1 − 3(1 − *F*)^2/3^ + 2(1−*F*) = −0.0852 + 0.2460*t*	0.2460	0.9876
	chemical reaction	1 − (1 − *F*)^1/3^ = −0.024 + 0.1648*t*	0.1648	0.9882
Ce^3+^	liquid film diffusion	−ln(1 − *F*) = −0.4764+0.9195*t*	0.9195	0.9835
	particle diffusion	1 − 3(1 − *F*)^2/3^ + 2(1 − *F*) = −0.1495 + 0.2213*t*	0.2213	0.9922
	chemical reaction	1 − (1 − *F*)^1/3^ = −0.0386 + 0.1915*t*	0.1915	0.9762
Y^3+^	liquid film diffusion	−ln(1 − *F*) = −0.6299 + 1.4323*t*	1.4323	0.9887
	particle diffusion	1 − 3(1 − *F*)^2/3^ + 2(1 − *F*) = −0.1746 + 0.3341*t*	0.3341	0.9952
	chemical reaction	1 − (1 − *F*)^1/3^ = −0.0288 + 0.2543*t*	0.2543	0.9854

**Table 2:** Langmuir and Freundlich adsorption isotherm parameters.

Metal Ions	Langmuir			Freundlich		
	*K*_L_ (L·mol^−1^)	*Q*_m_ (mmol·g^−1^)	*R* ^2^	*K*_F_	*n*	*R* ^2^
La^3+^	295.7	2.183	0.9941	0.0098	2.548	0.9904
Ce^3+^	634.9	2.592	0.9991	0.0120	2.674	0.9735
Y^3+^	596.1	2.884	0.9995	0.0140	2.721	0.9158

**Table 3:** Thermodynamics parameters for La(III), Ce(III), and Y(III) adsorption by resin.

Metal Ions	∆*H* (KJ·mol^−1^)	∆*G* (KJ·mol^−1^)	∆*S* (J·mol^−1^·K^−1^)
La^3+^	9.777	−6.631	52.42
Ce^3+^	21.76	−6.958	91.75
Y^3+^	17.35	−7.081	78.05

**Table 4:** Results of desorption efficiency.

Metal Ions	E_1_ (%)	E_2_ (%)	E_3_ (%)
La^3+^	74.2	89.3	98.4
Ce^3+^	76.6	90.6	99.1
Y^3+^	78.1	91.4	98.8

**Table 5:** Comparison of maximum adsorption capacity of Ce(III) on various adsorbents.

Absorbent	Q_max_ (mmol·g^−1^)	Reference
polystyrene-poly(hydroxamic acid) resin	1.530	This work
Dowex 50WX8	0.169	[42]
Lewatit MDS 200H	0.173	[42]
RCAC	0.420	[43]
P(HA–SSS)	0.564	[26]
poly(styrene-p-hydroxamic acid)	0.620	[11]
CIP	0.932	[44]
D151	2.800	[37]
